# 

**Published:** 1845-03

**Authors:** 


### CONTENTS

OF THE

### MEDICAL EXAMINER.

NEW SERIES—NO. III.—MARCH, 1845.

### ORIGINAL COMMUNICATIONS.

Northern Schools and Southern Medicine, - - . - - - 137

Contribution to the History of Malaria. In a letter from Dr. R. S. Holmes, of the Medical Staff of the United States Army, to Pro. fessor Dunglison. (Communicated by the latter,) - - - 144

Account of a Case in which sixteen sewing needles and four pins were extracted from the Wrist. By H. Gordon P. Spencer, Student of Medicine, of Champion, Jefferson co., N. Y. - - - 146

On Strictures of the Rectum. By Thomas D. Mütter, M. D., Professor of Surgery in Jefferson Medical College, Philadelphia,- - 148

Malignant Stricture, - - - - - - - - - - 148

Treatment, - - - - - - - - - - 152

Excision of the Rectum, - - - - - - - - 154

The Establishment of an Artificial Anus, - - - - - 156

Operation as performed by Amussat, - - - - - 158

Operation of Ashmead, - - - - - - - 159

### CLINICAL LECTURES AND REPORTS.

### PHILADELPHIA HOSPITAL,

*Saturday, December* 21, 1844.

Clinic of Professor Dunglison.

Scrofulosis, - - - - - - - - - - - - 162

Death from Drowning, - - - - - - - - - - 168

### BIBLIOGRAPHICAL NOTICES.

De l'Identité du Typhus et de la Fièvre Typhoïde; par C. E. S. Gaultier de Claubry, Chevalier de l'Ordre Royal de la Légion de l'Honneur, Docteur en Médecine et Agrégé libre de la Faculté de Paris, &c. &c. - - - - - - - - - - - - - 172

New Elements of Operative Surgery. By Alf. A. L. M. Velpeau, Professor of Surgical Clinique of the Faculty of Medicine of Paris, &c. Carefully revised, entirely remodelled, and augmented with a treatise on minor Surgery. Illustrated by over 300 engravings, incorporated with the text; accompanied with an Atlas in quarto of twenty-two plates, representing the principal operative processes, surgical instruments, &c. First American, from the last Paris edition. Translated by P. S. Townsend, M. D., late Physician to the Seamen's Retreat, Staten Island, New York. Augmented with the addition of several hundred pages of entirely new matter, comprising all the latest improvements and discoveries in Surgery in America and Europe, up to the present time. Under the supervision of, and with notes and observations by Valentine Mott, M. D., &c. - - - - - 175

The Anatomy and Philosophy of Expression as connected with the Fine Arts. By Sir Charles Bell, K. H. Third Edition Enlarged, - 179

Annual Oration delivered before the Philadelphia Medical Society, by appointment, at the opening of its Session of 1844–5. By D. Francis Condie, M. D., Honorary Member of the Society, - 181

An Essay on Curvatures and Diseases of the Spine, including all the remote or spinal distortions: to which the Fothergillian Gold Medal was awarded by the Medical Society of London. By R. W. Bampfield, Esq., one of the Surgeons to the Royal Metropolitan Infirmary for Diseases of Children, etc. etc. Edited by J. K. Mitchell, M. D., Professor of the Practice of Medicine in the Jefferson Medical College of Philadelphia, etc. etc. - - 184

An Introductory Lecture on the Means of Promoting the Intellectual Improvement of the Students and Physicians of the Valley of the Mississippi. Delivered in the Medical Institute of Louisville, November 4th, 1844. By Daniel Drake, M. D., Professor of Pathology and the Practice of Medicine, - - - - - - 188

Hints on the re-organization of the Navy, including an Examination of the claims of its Civil Officers to an Equality of Rights, - - 188

The Cyclopædia of Practical Medicine. By Doctors Forbes, Tweedie and Conolly. Edited by Professor Dunglison. Part XXIV. Containing also the title page and index, - - - - 189

A Dictionary of Practical Medicine; comprising General Pathology, the Nature and Treatment of Diseases, Morbid Structures, &c. By James Copland, M. D., F. R. S. Edited, with additions, by Charles A. Lee, M. D. Part III. - - - - - - - - 189

### EDITORIAL.

Clinical Class at Blockley Hospital, - - - - - - - - 190

Medical Classes, - - - - - - - - - - 190

### RECORD OF MEDICAL SCIENCE.

Medical Society of London, - - - - - - - - - - 191

Case of Hobnailed Liver, - - - - - - - - 191

Extensive Scrofulous Disease, - - - - - - - - 192

Intestinal concretion, - - - - - - - - - - - - 193

Aneurism of the Abdominal Aorta, - - - - - - - 193
The Therapeutics of Nature, - - - - - - - - 194
On the Occasional Pulsation of the Veins, - - - - - - - 194
On the use of the Thymus Gland, - - - - - - - - 196
Spontaneous Expulsion of the Os Hyoides, - - - - - - 196
Surgical Memoranda by M. Vidal, - - - - - - - - 197
Remarks on Ambulant or Erratic Erysipelas, - - - - - 197
Case of Copper-Coloured Syphilitic Eruption affecting the Conjunctiva. By Alfred Smee, F. R. S. - - - - - - - -
Case of sudden blindness, (produced by effusion of blood on the optic nerve? - - - - - - - - - - 200

